# *α*-Amylase Inhibitory Activity of *Catunaregam spinosa* (Thunb.) Tirveng.: *In Vitro* and *In Silico* Studies

**DOI:** 10.1155/2021/4133876

**Published:** 2021-12-13

**Authors:** Deepak Timalsina, Deepti Bhusal, Hari Prasad Devkota, Krishna Prasad Pokhrel, Khaga Raj Sharma

**Affiliations:** ^1^Central Department of Chemistry, Tribhuvan University, Kirtipur, Kathmandu 44618, Nepal; ^2^Graduate School of Pharmaceutical Sciences, Kumamoto University, 5-1 Oe-honmachi, Chuo-Ku, Kumamoto 862-0973, Japan

## Abstract

*α*-Amylase is an enzyme involved in the breaking down of large insoluble starch molecules into smaller soluble glucose molecules. *Catunaregam spinosa* (Thunb.) Tirveng. (syn. *Randia dumetorum* (Retz.) Lam., Family: Rubiaceace) has been used as traditional medicine for the treatment of gastrointestinal problems, skin diseases, and diabetes. In this context, we studied the *in vitro α*-amylase inhibiting properties of methanol extracts of leaves and bark of *C. spinosa*. The methanol extract of bark was further fractionated into hexane, dichloromethane and ethyl acetate, and water-soluble fractions, and their *α*-amylase inhibitory activity was evaluated. *In silico* molecular docking and ADMET analysis of several compounds previously reported from the bark of *C. spinosa* were also performed. The *in vitro α*-amylase inhibition activity assay of the dichloromethane fraction of extract of bark (IC_50_: 77.17 ± 1.75 *μ*g/mL) was more potent as compared to hexane and ethyl acetate fractions. The *in silico* molecular docking study showed that previously reported compounds from the stem bark such as balanophonin, catunaregin, *β*-sitosterol, and medioresinol were bounded well with the active catalytic residue of porcine pancreatic *α*-amylase indicating better inhibition. The ADMET analysis showed the possible drug-likeness and structure-activity relationship of selected compounds. These compounds should be studied further for their potential *α*-amylase inhibition in animal models.

## 1. Introduction

The *α*-amylase is a prominent enzyme found in saliva and pancreatic juice which helps to break down large insoluble starch molecules into glucose that is absorbable by the digestive system [1]. The inhibitors of *α*-amylase help to delay the breakdown of starch into glucose molecules [2]. In patients with hyperglycemia, an effective treatment option could be the inhibition of pancreatic *α*-amylase that controls carbohydrate absorption [3]. The clinically available inhibitor, i.e., acarbose, has several side effects associated with gastrointestinal problems such as flatulence and diarrhea [4]. Various traditional medicines and herbs are well known for their role in the prevention and treatment of diabetes. Some plant-derived constituents with antidiabetic properties have been isolated and shown to have high potential and lower side effects than synthetic drugs [5].

**Figure 1:**
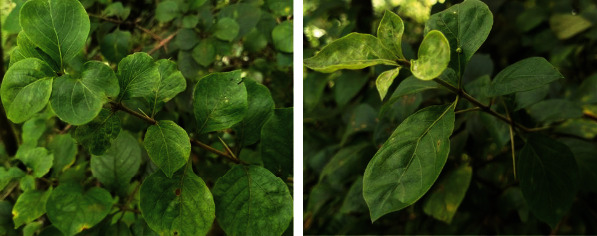
Photographs of *Catunaregam spinosa* (Thunb.) Tirveng.

*Catunaregam spinosa* (Thunb.) Tirveng. (Syn. *Randia dumetorum* (Retz.) Lam., Family: Rubiaceae) (Figure 1) occurs at around 1000-1500 m altitude from the sea level [6] and is distributed in the tropical and semitropical climatic zones [7]. This plant has been reported from various parts of China, Nepal, Bangladesh, and India [8]. Traditionally, it has been used to treat various symptoms such as skin diseases, tumors, wounds, ulcers, and diabetes [6, 9]. In India, the bark juice of this plant is used to treat several gastrointestinal problems such as diarrhea and dysentery [10]. In the traditional Ayurvedic system, this plant is used in the treatment procedure called “Vamana” to prevent forthcoming diseases such as hyperacidity, rhinitis, migraine, and anorexia [11]. The wide variety of bioactive compounds such as triterpene saponins [12–14], iridoid [15], flavonoids [16], and dihydroisocumarins [7] are reported which have shown potent an anti-inflammatory, antioxidant [17], antidiabetic, and antihyperlipidemic [18] activities. In this study, we evaluated the *in vitro α*-amylase inhibitory activity of the extracts of leaves and bark of *C. spinosa* and fractions of bark extract. To understand the mechanism, the *in silico* analysis of the previously reported compounds from the bark was performed via molecular docking studies and ADMET analysis. This study can be useful to justify the ethnomedicinal use of *C. spinosa* in diabetes-related problems. This can pave the way towards the discovery of a plant-based *α*-amylase inhibitor from *C. spinosa*.

## 2. Materials and Methods

### 2.1. Chemical Reagents

The substrate 2-chloro-4-nitrophenyl-*α*-D-maltotrioside (CNPG3), enzyme porcine pancreatic *α*-amylase (PPA), and acarbose were purchased from Sigma-Aldrich, Germany. All other reagents were of analytical grade.

### 2.2. Plant Identification, Processing, and Extraction

The leaves and bark of *C. spinosa* were collected from the upper region of Sindhupalchok district, Nepal, and identified at the National Herbarium and Plant Laboratories (KATH) of the Department of Plant Resources, Ministry of Forest and Environment, Kathmandu, Nepal (Voucher code: DT 01). The collected materials were shade dried and grounded to get powder. The powdered leaves and bark (800 g each) were extracted in methanol separately by maceration for 72 hours. The extracts were then filtered and dried by using a rotary evaporator.

### 2.3. Fractionation

**Figure 2:**
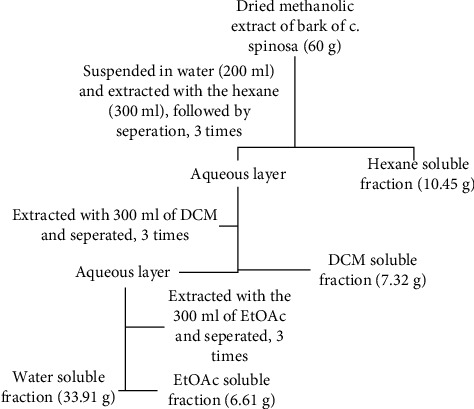
Schematic diagram of fractionation of methanolic bark extract in different solvent.

The more active extract, i.e., extract of the bark, was selected for fractionation using different solvents hexane, dichloromethane (DCM), and ethyl acetate (EtOAc) based on polarity. The methanolic extract was suspended in 200 mL of water and extracted with hexane. The aqueous layer was again extracted with dichloromethane followed by ethyl acetate. The process was repeated three times for each solvent as shown in Figure 2. The fractions were dried using a rotary evaporator [19, 20]. The methanol extracted crude extract, and solvent fractions were employed for the *in vitro α*-amylase inhibition experiment.

### 2.4. Determination of *α*-Amylase Inhibition

The *α*-amylase activity measurements were performed at 37°C using 2-chloro-4-nitrophenyl *α*-D-maltotroside (CNPG3) as a substrate [21]. The *α*-amylase hydrolyzes the CNPG3 substrate into 2-chloro-4-nitrophenyl *α*-D-maltotroside, 2-chloro-4-nitrophenol, maltrioside, and glucose. The substrate was prepared in 50 mM phosphate buffer (pH 6.95, containing 6.7 mM NaCl) and diluted to maintain the 375 *μ*M concentration. The *α*-amylase was prepared and diluted to 1.5 units/mL by dissolving in the same buffer. Plant extracts were dissolved in DMSO by using a vortex machine and prepared 5000-50 *μ*g/mL by serial dilution. The positive control acarbose was also prepared in the same way.

The 80 *μ*L of *α*-amylase solution (1 *μ*g/mL in phosphate buffer at pH 6.9) was added to 20 *μ*L of plant sample, positive control, and negative control, respectively, in triplicate. The reaction mixture was then incubated at 37°C for 15 min and the initial reading was taken by measuring absorbance at 405 nm. After 15 min, the 100 *μ*L substrate was added to the reaction mixture of sample and *α*-amylase. The progress of reaction was monitored by measuring the release of 2-chloro-4-nitrophenol spectrophotometrically. The absorbance was measured at 405 nm in a 96-well plate (Synergy LX, BioTek, Instruments, Inc., USA) The inhibition of methanolic crude extract, hexane fraction, dichloromethane fraction, and ethyl acetate fraction from the bark was recorded. The enzyme inhibition was calculated by the formula:

(1) $$\begin{array}{c} \%\text{ inhibition}=\frac{\left(\text{Absorbance of control}-\text{Absorbance of sample}\right)}{\text{Absorbance of control}}*100\%. \end{array}$$

The IC_50_ value was calculated by using software “GraphPad Prism.”

### 2.5. Molecular Docking Studies

#### 2.5.1. Determination of Ligands and Receptors

**Figure 3:**
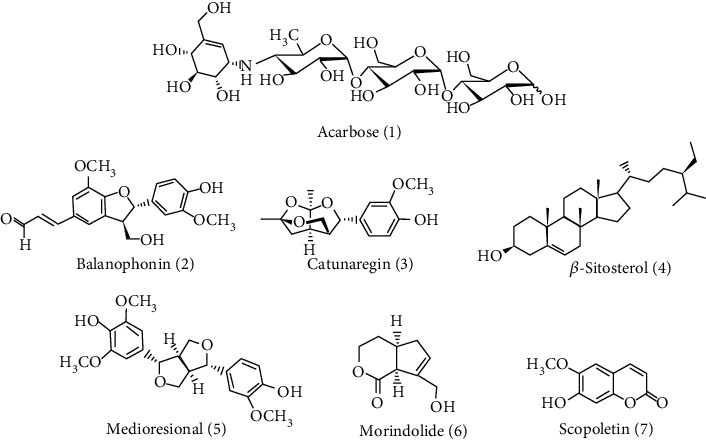
Chemical structures of acarbose and selected compounds for molecular docking.

The library of more than 37 known isolated compounds from the stem bark of *C. spinosa* was prepared from the extensive literature survey. The bioactive compounds were of different classes such as lignans, coumarins, isocoumarins, and saponins. The compounds were selected based on their molecular weight (<500 g/mol) and previous *in vitro* and *in silico* studies [22]. These compounds were balanophonin (**2**) [7], catunaregin (**3**) [23, 24], *β*-sitosterol (**4**) [25], medioresinol (**5**), morindolide (**6**), and scopoletin (**7**) [7], which are listed with their physicochemical properties in Table 1, and their chemical structures are presented in Figure 3.

The structure of the selected compounds along with the standard acarbose was either drawn in the ChemDraw and was converted to 3D or downloaded from the PubChem database. Finally, the structures were transformed to a .pdb file format, which is readable by BIOVIA Discovery Studio.

Compared to other amylases, the homology modeling of porcine and human pancreatic *α*-amylase is very similar at about 87.1% [28, 29]. The porcine pancreatic *α*-amylase (PDB ID:1OSE) was chosen for the molecular docking analysis. The three-dimensional structure of the protein was obtained from the Protein Data Bank (PDB) in the .pdb file format [22].

#### 2.5.2. Ligand and Receptor Preparation

The protein complexed with acarbose was downloaded from the Protein Data Bank (http://www.rcsb.org/pdb) and prepared using BIOVIA Discovery Studio Visualizer 2020 [22]. The water, ligands, and other heteroatoms were removed, and polar hydrogen was added after defining the binding site of the already bound ligand [30]. The attributes were noted before removing the already complex ligand.

The ligand was downloaded from the PubChem database (http://pubchem.ncbi.nlm.nih.gov). They were optimized for docking by adjusting the torsion tree and were saved in the .pdbqt file format using the AutoDock tool [31].

#### 2.5.3. Determination of Active Sites

The active site was determined by several bases such as the interacting site of already complexed (cocrystallized) ligands [32], the sites reported from similar previous studies as well as blind docking. The grid box was prepared by using the AutoDock tool, and the receptor grid center was placed in the receptors' active site residue. The amino acid determined in the active site was used to evaluate the result of our docking study. For the blind docking, the grid box was made maximum to cover the whole part of the receptor, allowing the ligand to be docked in all parts of the receptor [33].

#### 2.5.4. Molecular Docking

**Figure 4:**
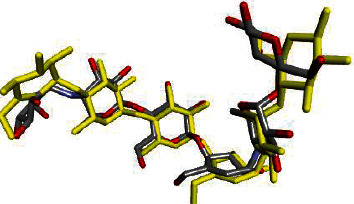
Superimposing of cocrystallized ligands extracted (yellow) and redocked ligand (grey-red) with RMSD value < 1 Å.

Molecular docking studies were performed by using AutoDock Vina [31]. The configuration file was generated defining the attributes such as evaluation of grid box coordinates and size. The obtained grid box was as follows: *X* = 35.661954, *Y* = 37.373877, and *Z* = −1.203215; and the size was *X* = 30, *Y* = 30, and *Z* = 30, energy range = 3, and exhaustiveness = 8. The output file in the .pdbqt format and the log file in the .txt format were written in the configuration file. The docking studies were validated by the superimposing of cocrystallized ligands (Figure 4) extracted from cocrystals and redocked with the same 1OSE receptor with its RMSD value < 2 Å [34].

The studies were performed for the selected compounds along with the standard acarbose. After docking the best pose with the lowest B.E. (Kcal/mol) and the highest number of H-bonding, it was selected for further visualization. The binding interactions between ligands and receptors were visualized by the BIOVIA Discovery Studio Visualizer.

### 2.6. Pharmacokinetic and ADMET Profile

In the pharmaceutical industry, drug-likeness, ADMET, and target profiles of potential hit compounds are critical in reducing side effects [35]. In the current analysis, the pharmacokinetic properties were predicted by web-based application swissADME (https://www.swissadme.ch) [36]. The drug-likeness properties are determined by the rule of five, also called Lipinski's rule. When there are more than 5 H-bond donors and 10 H-bond acceptors, the molecular weight (MWT) is greater than 500, and the measured Log *P* (CLog*P*) is greater than 5 (or Mlog*P* > 4.15); weak absorption or permeation is more possible [37, 38]. This idea is related to the SLIPPER-2001 program, which employs physicochemical descriptors and molecular similarities to forecast properties like lipophilicity, solubility, and fraction absorbed in humans [39]. The toxicity analysis was done by a similar web-based application ProTox-II [40]. Several parameters such as bioavailability, brain penetration, oral absorption, carcinogenicity, immunotoxicity, and human intestinal absorption properties were calculated for all active compounds. Toxicity is important to consider while developing drugs because it aids in assessing the toxic dosage in animal model experiments and reduces the number of animal model studies [41, 42].

## 3. Results

### 3.1. *α*-Amylase Inhibition

The extracts of leaves and bark of *C. spinosa* were both potent towards *α*-amylase inhibition activity. Among the fractions of bark extract, dichloromethane and ethyl acetate fractions showed good *α*-amylase inhibition activity in the screening result at 500 *μ*g/mL and hexane fraction was less potent.

**Figure 5:**
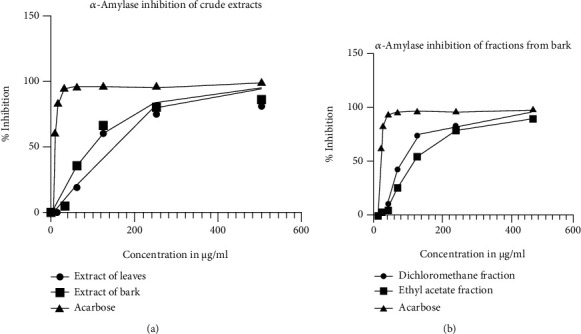
A plot for *α*-amylase inhibition of (a) crude extracts and (b) acarbose and extracts from bark and acarbose.

The graphical presentations (Figure 5) for inhibition and IC_50_ values of all extracts and fractions were compared to that of acarbose.

The graphical trend of % inhibition against concentration was similar to that of standard acarbose indicating *α*-amylase inhibition activity of the giving extract. The IC_50_ values obtained for the extracts are tabulated in Table 2.

The crude methanolic extract of leaves had a higher IC_50_ value of 119.7 ± 2.79 *μ*g/mL than that of crude bark extract, 94.66 ± 2.19 *μ*g/mL showing lower potency. The hexane fraction showed very less inhibition even at the higher dose. The DCM fraction and ethyl acetate fraction had an IC_50_ value of 77.17 ± 1.75 and 116 ± 1.60 *μ*g/mL, respectively, compared to the standard acarbose whose IC_50_ was 6.34 ± 0.07 *μ*g/mL.

### 3.2. Molecular Docking Analysis

The careful investigation of cocrystallized ligand interaction BIOVIA Discovery Studio revealed that the catalytic residue contains H-bonding with ASP-300, GLU-233, GLY-306, HIS-299, HIS-305, ASP-197, HIS-101, TRP-59, GLN-63, VAL-163, GLY-164, SER-105, GLY-106, and LYS-200 which is similar to that of previously reported active residue interacting with the acarbose, a competitive inhibitor of *α*-amylase [43].

The results of docking score met well with the *in vitro* analysis. The binding affinity (docking score) and H-bonding catalytic residue for all selected compounds against porcine pancreatic *α*-amylase (PDB ID: 1OSE) are presented in Table 3.

**Figure 6:**
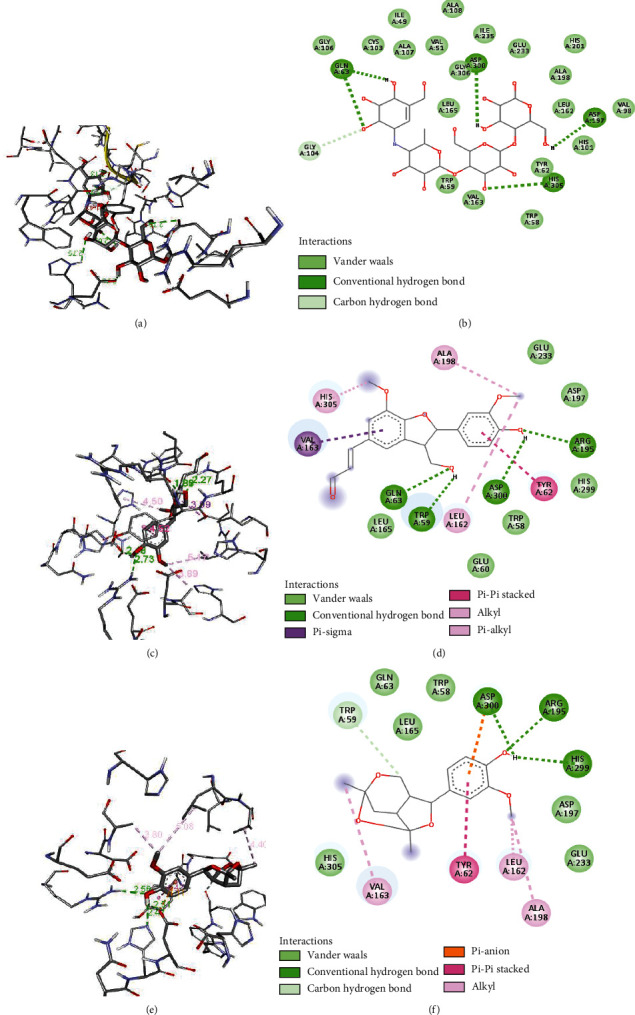
3D (a, c, and e) and 2D (b, d, and f) interactions of protein and (a, b) acarbose, (c, d) balanophonin, and (e, f) catunaregin.

**Figure 7:**
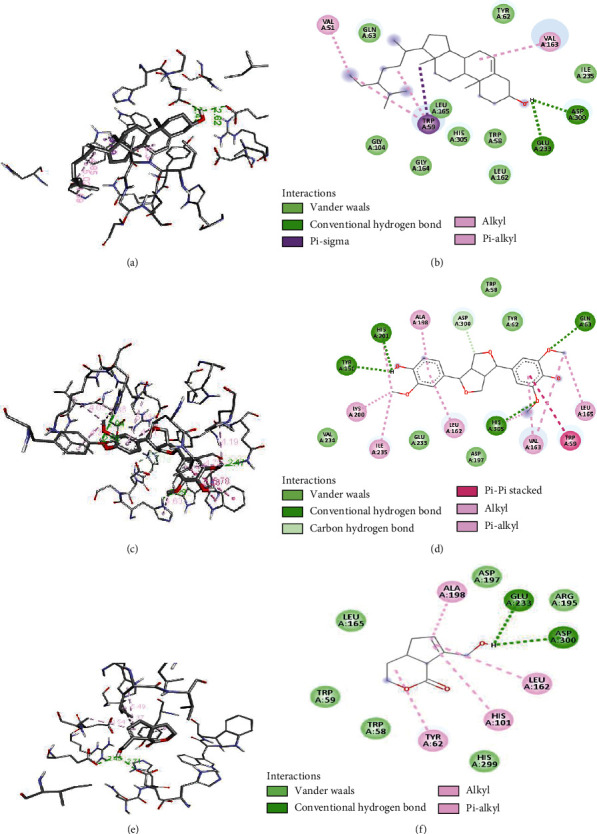
3D (a, c, and e) and 2D (b, d, and f) interactions of protein and (a, b) *β*-sitosterol, (c, d) medioresinol, and (e, f) morindolide.

**Figure 8:**
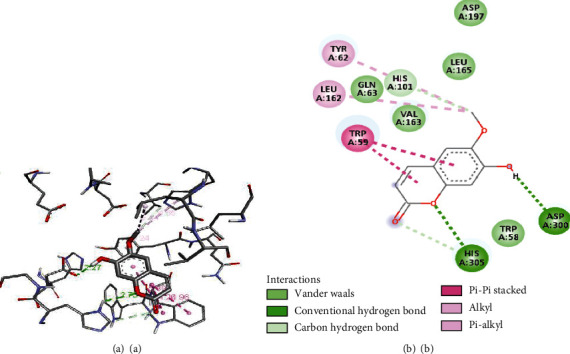
3D (a) and 2D (b) interactions of protein and (a, b) scopoletin.

Several other interactions such as pi-pi, van der Waals, and hydrophobic interactions occurred in between the ligands and receptors, whose detailed visualization is presented in Figures 6–8.

### 3.3. Pharmacokinetic and ADMET Properties

The pharmacokinetics and drug-likeness properties of the docked compounds are tabulated in Table 4. The polar surface area has been widely used as a descriptor of drug transport properties such as intestinal absorption and blood-brain barrier penetration. It is also the number of polar atoms like oxygen, nitrogen, and their attached hydrogens' contributions to the molecular (usually van der Waals) surface region [44]. The standard compound, acarbose (**1**) violates three rules out of five and rules out from being drug-likeness. Compound **5** violates one rule out of five while all rest compounds were found with no violations indicating better drug-like properties.

The result of ADME analysis by swissADME and toxicity analysis by ProTox-II is tabulated in Table 5.

## 4. Discussion

*Catunaregam spinosa* has been reported for its activity against diabetes in several ethnomedicinal studies; however, the detailed study on *α*-amylase inhibition has not been carried out to relate the antidiabetic properties. The extracts and compounds present in *C. spinosa* have not been studied in detail *in vitro* and *in silico* studies. The comparative *α*-amylase inhibiting properties of plant extracts and fractions revealed that the DCM fraction showed more potent behavior than the other fractions. The *in vitro α*-amylase inhibition analysis was reported for the first time from this plant. This can be related to the previously reported *in vivo* analysis of the antihyperglycemic activities of methanolic extract of *C. spinosa* fruit extract in the nicotinamide and streptozotocin-induced male Wistar diabetic rats of which *C. spinosa* fruit extract (400 mg/kg) decreased the blood sugar level from 106.16 ± 7.8 to 87.5 ± 5.00 mg/dL [18] and in stem bark extract (400 mg/kg) treated group to 94.00 ± 1.577 mg/dL as compared to the diabetic control group [9].

The evaluated docking score and visualization of interactions suggest that the binding affinity and number of H-bonding with the catalytic site of the receptor of selected compounds were comparable. Hydrogen bonding interactions with receptors are essential as they have the organization needed for distinct folding and selectivity which supports the molecular recognition at the ligand-protein interface [45]. Out of the six selected, four compounds **2**, **3**, **4**, and **5** had strong interaction (ΔG ≤ −8.0 kcal/mol) with the receptor. The highest docking score was for compound **4** but with only two H-bonding with active site residue (ASP-300, GLU-233), while compounds **2** and **5** have four H-bonding with active site (ARG-195, ASP-300, TRI-59, and GLN-63) and (HIS-201, TYR-151, HIS-305, and GLN-63), respectively, similar to that of standard compound **1** with H-bonding (ASP-300, HIS-305, ASP-197, and GLN-63). The compounds **6** and **7** have lower binding affinity and a lower number of H-bonding indicating weak interactions. The tested compound **4** has already been studied for its *in vivo* antidiabetic properties in streptozotocin-induced diabetic rats and was found to be effective by 78-100% to lower the blood glucose level [46]. The result showed that except for compounds **1** and **4**, all other compounds absorb well in the intestine. Cytochrome CYP450 (1A2, 2C9, 2C19, 2D6, and 3A4), which is primarily responsible for the biotransformation of more than 90% of drugs in phase-1 metabolism, plays a significant role in drug metabolism [47]. Compounds **3** and **5** showed CYP2D6 inhibition, and compound **7** was found to inhibit CYP1A2. Compounds **3**, **6**, and **7** readily cross the BBB while the rest of the compound was not found to cross the BBB. The toxicity of docked compounds suggests that compounds **1-5** are immune toxic while **6** is not immune toxic. None of the compounds were shown to have hepatotoxicity and cytotoxicity. The toxicity class and predicted LD_50_ suggest that compound **1** was much safer to use; compounds **2**, **6**, and **7** were also found to be safer to use than the rest of the compounds.

## 5. Conclusion

Using *in vitro* analysis, we found that the dichloromethane fraction fractionated from the methanolic extract of bark of *C. spinosa* has a high potential of *α*-amylase inhibition. The fact was supported by a molecular docking study of porcine pancreatic *α*-amylase protein and various known compounds from the bark of *C. spinosa*. The interactions with the catalytic sites of the protein and the selected compounds were similar to the interaction of the standard *α*-amylase inhibitor, acarbose. We have also studied the drug-likeliness of selected compounds and ADMET analysis which shows that the selected compounds follow the drug properties. In the present study, compounds **2**, **3**, and **5** score high with the lowest binding affinity and a higher number of H-bonding towards the catalytic residue. Thus, the isolation of high-scoring compounds **2**, **3**, and **5** and their *in vivo* animal study for antihyperglycemic and antidiabetic properties could further confirm the potential of this compound acting as an antidiabetic drug. This study could be helpful to select the compounds for animal study and increase the efficiency of clinical wet-lab studies.

## Figures and Tables

**Table 1:** Physicochemical properties of selected compounds.

S.N.	Compound	PubChem CID	Molecular weight (g/mol)	Molecular formula	References
1.	Acarbose	41774	645.6	C_25_H_43_NO_18_	[26]
2.	Balanophonin	23252258	356.4	C_20_H_20_O_6_	[7, 27]
3.	Catunaregin	45258336	292.33	C_16_H_20_O_5_	[23, 24]
4.	*β*-Sitosterol	222284	414.7	C_29_H_50_O	[25]
5.	Medioresinol	181681	388.4	C_21_H_24_O_7_	[7, 27]
6.	Morindolide	10397184	168.19	C_9_H_12_O_3_	[7, 27]
7.	Scopoletin	5280460	192.17	C_10_H_8_O_4_	[7, 27]

**Table 2:** IC_50_ values of plant extracts, fractions, and standard acarbose.

Plant extract	IC_50_ (*μ*g/mL, mean ± SEM)
Extract of leaves	119.70 ± 2.79
Extract of bark	94.66 ± 2.19
Dichloromethane fraction of extract of bark	77.17 ± 1.75
Ethyl acetate fraction of extract of bark	116.00 ± 1.60
Acarbose	6.34 ± 0.07

**Table 3:** Docking score results for porcine pancreatic amylase (PDB ID: 1OSE) receptor and selected ligands.

S.N.	Ligand	Docking score (kcal/mol)	Binding features (H-bond length in Å) with active site residue
1.	Acarbose	-8.0	ASP-300 (2.40 Å), HIS-305 (2.73 Å), ASP-197 (2.70 Å), GLN-63 (2.07 Å)
2.	Balanophonin	-8.3	ARG-195 (2.73 Å), ASP-300 (2.48 Å), TRI-59 (2.27 Å), GLN-63 (2.27 Å)
3.	Catunaregin	-8.1	ASP-300 (2.51 Å, 4.11 Å), HIS-299 (2.47 Å), ARG-195 (2.56 Å)
4.	*β*-Sitosterol	-8.9	ASP-300 (2.45 Å), GLU-233 (2.62 Å)
5.	Medioresinol	-8.5	HIS-201 (2.94 Å), TYR-151 (2.88 Å), HIS-305 (2.29 Å), GLN-63 (2.47 Å)
6.	Morindolide	-6.0	GLU-233 (2.45 Å), ASP-300 (2.71 Å)
7.	Scopoletin	-6.3	ASP-300 (2.27 Å), HIS-305 (2.73 Å)

**Table 4:** Pharmacokinetic and drug-likeness properties of selected ligands.

S.N.	Name	No of rotatable bonds	TPSA1	Consensus log *P*	LogS (ESOL2)	Drug-likeness (Lipinski's rule)
1.	Acarbose	9	321.17 Å	-6.22	2.13	No (3 violations)
2.	Balanophonin	6	85.22 Å	2.34	-3.28	Yes (0 violations)
3.	Catunaregin	2	57.15 Å	2.09	-2.88	Yes (0 violations)
4.	*β*-Sitosterol	6	20.23 Å	7.19	-7.90	Yes; (1 violations)
5.	Medioresinol	5	86.61 Å	2.33	-3.65	Yes (0 violations)
6.	Morindolide	1	46.53 Å	0.86	-0.82	Yes (0 violations)
7.	Scopoletin	1	59.67 Å	1.52	-2.46	Yes (0 violations)

TPSA: topological polar surface area; ESOL: estimated aqueous solubility; data source: https://www.swissadme.ch.

**Table 5:** ADMET profiles of selected compounds.

S.N.	Compounds	GI Abs.	P-gp substrate2	BBB permeation	CYP3 inhibition	Hepatotoxicity	Cytotoxicity	Immune toxicity	Toxicity class	Predicted LD50 (mg/kg)
1.	Acarbose	Low	Yes	No	No	—	No	Yes	6	24000
2.	Balanophonin	High	Yes	No	No	No	No	Yes	5	5000
3.	Catunaregin	High	Yes	Yes	Yes (CYP2D6)	No	No	Yes	4	600
4.	*β*-Sitosterol	Low	No	No	No	No	No	Yes	4	890
5.	Medioresinol	High	Yes	No	Yes (CYP2D6)	No	No	Yes	4	1500
6.	Morindolide	High	No	Yes	No	No	No	No	5	5000
7.	Scopoletin	High	No	Yes	Yes (CYP1A2)	—	No	—	5	3800

## Data Availability

The data sets to support the findings of this study are available from the corresponding author and submitting author upon request.

## Abbreviations

*μ*g/mL: Micro grams per millilitre
ADMET: Absorption distribution, metabolism excretion toxicity
BBB: Blood-brain barrier
CNPG3: 2-Chloro-4-nitrophenyl-*α*-D-maltotrioside
DCM: Dichloromethane
DMSO: Dimethyl sulphoxide
IC_50_: Inhibition concentration at 50%
Kcal/mol: Kilocalorie per mol
LD_50_: Lethal concentration at 50%
mg/mL: Milligram per millilitre
nm: Nanometer
PDB: Protein Data Bank
PPA: Porcine pancreatic *α*-amylase
RMSD: Root mean square deviation
TPSA: Topological polar surface area
*μ*L: Microlitre.
